# Place of death in the Czech Republic and Slovakia: a population based comparative study using death certificates data

**DOI:** 10.1186/1472-684X-13-13

**Published:** 2014-03-20

**Authors:** Martin Loucka, Sheila A Payne, Sarah G Brearley

**Affiliations:** 1 The International Observatory on End-of-Life Care, Division of Health Research, Faculty of Health and Medicine, Lancaster University, Lancaster LA1 4YG, UK

**Keywords:** Health policy, End-of-life care, Palliative care, Location of death, Eastern Europe

## Abstract

**Background:**

Place of death represents an important indicator for end-of-life care policy making and is related to the quality of life of patients and their families. The aim of the paper is to analyse the place of death in the Czech Republic and Slovakia in 2011. Research questions were focused on factors influencing the place of death and specifically the likelihood of dying at home.

**Methods:**

Whole population data from death certificates for all deaths in the Czech Republic and Slovakia in 2011 were used for bivariate and multivariate analyses. Separate analysis using binary logistic regression was conducted for subpopulation of patients who died from chronic conditions.

**Results:**

The majority of population in both countries died in hospitals (58.4% the Czech Republic, 54.8% Slovakia), less than one-third died at home. In case of chronic conditions, death at home was significantly associated with underlying cause of death (cancer and heart failure), being male, age (older than 85, Slovakia only) and higher education (the Czech Republic only). Cancer and heart failure patients had higher chances to die at home than other chronic conditions.

**Conclusions:**

Czech and Slovak patients with chronic conditions are more likely to die in hospitals than in some other European Union member countries. This finding should be addressed by policy makers in promoting home hospice care services and education in palliative care for staff in nursing homes and other end-of-life settings.

## Background

Death certificates represent a useful monitoring tool for public health policy [1,2]. Choosing the best quality measures for end-of-life care is a very complex issue [3,4] and information about place of death has been suggested to be one of the key indicators [5-7]. Together with research on place of death preferences [8,9], the analysis of actual place of death is essential in planning appropriate end-of-life care policy [10]. Although the relationship between preference for place of death and actual choice is rather complex and influenced by various factors [11,12], dying at home is often cited as the indicator of quality of end-of-life care because home is usually the preferred place for most people [13]. Previous studies have also highlighted the association between place of death with health care expenditure [14], the quality of life of dying patients and the bereavement outcomes of their relatives [15].

The modern form of death certificates (List o prohlídce mrtvého) has been collected in the Czech Republic and Slovakia since 1964 [16]. Similar to other European Union countries, it is completed by the attending physician usually at the place of death. It consists of both an administrative and clinical section and is subsequently sent to national statistics offices for further processing. The national statistics offices use parts of death certificates to analyse and publish official mortality statistics. Data about place of death have been included in this official database since 2007 in the Czech Republic and since 2011 in Slovakia.

To our knowledge, this is the first study using the whole population death certificates data from the Czech Republic and Slovakia and also from the region of Eastern Europe. The study sought to answer the following research questions:

1) What was the general distribution of cause and place of death in the Czech Republic and Slovakia in 2011?

2) What was the distribution in place of death for deaths caused by chronic conditions in the Czech Republic and Slovakia in 2011?

3) What factors influence the likelihood that a person in the Czech Republic or Slovakia died from a chronic condition at home in 2011?

## Methods

### Study design

Death certificates data for all deaths in the Czech Republic (total population 10,505,445) and Slovakia (total population 5,404,322) in 2011 (N = 154288) were obtained from the Institute of Health Information and Statistics of the Czech Republic and the National Health Information Centre of the Ministry of Health of the Slovak Republic. They were received in anonymized form and collated together into one database. As the data were obtained in anonymized form and cannot be tracked back to individuals, ethical approval was not required.

Available variables were country, gender, age, date of death, education (elementary, secondary lower, secondary higher, university), marital status (single, married, divorced, widowed), cause of death (four-figure ICD-10 codes) and place of death. There is not a universally accepted coding system for place of death [1] and as such the information differed between both countries. In the Czech Republic the place of death categories include home, hospital, institutes for long term patients, social care homes, public space, during transportation to hospital and “other (please specify)”. In Slovakia, the categories are home, hospital, institutes for long term patients, public space, transport and “other (please specify)”. For the purpose of this study the datasets from both countries were merged into one database and categories of place of death were recoded to home, hospital, institutes for long term patients, and other (including all other options). The rationale for this recoding was that homes, hospitals, and institutes for long term patients included most of deaths in both countries (91% in CZ, 87.8% in SK) and the other categories are either marginal (public space and transportation with less than 4% of deaths) or not available from both countries (social care homes, available from CZ only with 5.3% of deaths).

### Statistical analysis

Descriptive statistics were used to present the basic distributions of variables. Bivariate analysis using *χ*^*2*^ Pearson tests were calculated in order to assess the associations between place of death and other variables. Separate analysis was conducted for deaths caused by chronic diseases as this sub-sample represents a population with similar end-of-life trajectory, potentially eligible for palliative care. We adopted the list of chronic diseases previously used in similar studies [2]. Statistical significance level was set as p < .01 with regard to the large sample size.

Significantly associated variables were later used in a binomial logistic regression model (enter selection procedure) comparing the chance of dying from chronic condition at home and in other settings in each country. The model was checked for multicollinearity and tested by Wald statistic and *χ*^2^ Pearson test. In order to obtain a consistent sample suitable for regression modelling only people older than 50 years of age were included (N = 68799), because age is strongly related with cause of death [6]. All analyses were executed in IBM SPSS Statistics version 20.

## Results

### General population

There were 102385 deaths in the Czech Republic (CZ) and 51903 in Slovakia (SK) in 2011. Mode for age of death was 82 in SK and 84 in CZ. The mean age of death was significantly lower in SK than in CZ (71.64 versus 74.07 years, p < .001). Men in both countries were significantly more likely to die at home than women, odds ratio 1.3 in CZ, 1.05 in SK. Major causes of death were diseases of circulatory system (around 50%) and neoplasms (around 25%). Distributions of deaths from specific ICD-10 categories are shown in Table 1.

**Table 1:** Deaths in the Czech Republic and Slovakia in 2011 by ICD-10 categories*

**ICD-10 category**		**Country**		**Total**
		**CZ**	**SK**	
I. Certain infectious and parasitic diseases	N	1319	414	1733
	%	1.3%	0.8%	1.1%
II. Neoplasms	N	26166	12071	38237
	%	26.2%	23.3%	25.2%
IV. Endocrine, nutritional and metabolic diseases	N	2634	714	3348
	%	2.6%	1.4%	2.2%
VI. Diseases of the nervous system	N	2013	763	2776
	%	2.0%	1.5%	1.8%
IX. Diseases of the circulatory system	N	49163	27306	76469
	%	49.2%	52.6%	50.4%
X. Diseases of the respiratory system	N	5396	3269	8665
	%	5.4%	6.3%	5.7%
XI. Diseases of the digestive system	N	4354	2870	7224
	%	4.4%	5.5%	4.8%
XIV. Diseases of the genitourinary system	N	1166	680	1846
	%	1.2%	1.3%	1.2%
XIX. Injury, poisoning and certain other consequences of external causes	N	5352	2821	8173
	%	5.4%	5.4%	5.4%
Other categories^a^	N	2375	995	3370
	%	2.4%	1.9%	2.2%
Total	N	99938	51903	151841
	%	100.0%	100.0%	100.0%

Missing CZ N = 2447, 1.6%.

*There was a significant association between ICD-10 category and country, tested by *χ*^2^ Pearson test, p < .001.

^a^III,V,VII,VIII,XII,XIII,XV-XVIII, each caused less than 1% of deaths.

There was a significant association between place of death and country (Table 2). When the place of death was recoded to binary variable (death at home or not), the odds of dying at home was 1.68 times higher in Slovakia (*χ*^*2*^ (1, N = 154288) = 1769.321, *p* < .001).

**Table 2:** Place of death in the Czech Republic and Slovakia in 2011*

		**Home**	**Hospital**	**Institutes for long term patients**	**Other**
**CZ**	N	20850	59767	12488	9280
	%	20.4%	58.4%	12.2%	9.1%
**SK**	N	15565	28451	1582	6305
	%	30.0%	54.8%	3.0%	12.1%

*There was a significant association between place of death and country, tested by χ^2^ Pearson test, p < .001.

There was a significant association between place of death and cause of death in both the Czech Republic (*χ*^*2*^ (27, N = 99938) = 9207.730, *p* < .001, Cramer’s *V* = .175) and Slovakia (*χ*^*2*^ (27, N = 51903) = 6446.631, *p* < .001, Cramer’s *V* = .203). The distributions are shown in Table 3.

**Table 3:** Cause of death and place of death in the Czech Republic and Slovakia in 2011*

		**Place of death**							
**Primary cause of death (ICD-10)**		**Home**		**Hospital**		**Institutes for long term patients**		**Other**	
		**CZ**	**SK**	**CZ**	**SK**	**CZ**	**SK**	**CZ**	**SK**
I. Certain infectious and parasitic diseases	N	32	14	1186	383	75	2	26	15
	%^a^	0.2%	0.1%	2.0%	1.3%	0.6%	.1%	0.3%	0.2%
II. Neoplasms	N	4391	3464	16719	7596	4375	337	681	674
	%	21.9%	22.3%	28.5%	26.7%	35.8%	21.3%	7.6%	10.7%
IV. Endocrine, nutritional and metabolic diseases	N	454	184	1737	452	253	25	190	53
	%	2.3%	1.2%	3.0%	1.6%	2.1%	1.6%	2.1%	0.8%
VI. Diseases of the nervous system	N	273	248	1030	348	482	49	228	118
	%	1.4%	1.6%	1.8%	1.2%	3.9%	3.1%	2.6%	1.9%
IX. Diseases of the circulatory system	N	11573	9748	26695	13050	5530	1008	5365	3500
	%	57.7%	62.6%	45.4%	45.9%	45.2%	63.7%	60.1%	55.5%
X. Diseases of the respiratory system	N	662	484	3847	2479	557	80	330	226
	%	3.3%	3.1%	6.5%	8.7%	4.6%	5.1%	3.7%	3.6%
XI. Diseases of the digestive system	N	532	462	3538	2287	181	26	103	95
	%	2.7%	3.0%	6.0%	8.0%	1.5%	1.6%	1.2%	1.5%
XIV. Diseases of the genitourinary system	N	75	90	968	549	81	15	42	26
	%	0.4%	0.6%	1.6%	1.9%	0.7%	0.9%	0.5%	0.4%
XIX. Injury, poisoning and certain other consequences of external causes	N	1440	768	1918	968	287	33	1707	1052
	%	7.2%	4.9%	3.3%	3.4%	2.3%	2.1%	19.1%	16.7%
other categories^b^	N	612	103	1103	339	400	7	260	546
	%	3.1%	0.7%	1.9%	1.2%	3.3%	.4%	2.9%	8.7%
Total	N	20044	15565	58741	28451	12221	1582	8932	6305
	%	100%	100%	100%	100%	100%	100%	100%	100%

CZ only: Missing N = 2447, 2.4% (Home 3.9%; Hospital 1.7%; Institutes 2.1%; Other 3.7%).

*There was a significant association between place of death and ICD-10 category in both countries, tested by *χ*2 Pearson test, p < .001.

^a^column percentages for each country.

^b^III,V,VII,VIII,XII,XIII,XV-XVIII, each caused less than 1% of deaths.

### Subpopulation of deaths from chronic conditions

#### Cause of death

Slightly less than half of all deaths in CZ and SK in 2011 were caused by chronic conditions with cancer and stroke being the most frequent diagnoses (see Table 4). There was a small significant difference between proportion of deaths by chronic conditions in the Czech Republic and Slovakia (*χ*^*2*^ (1, N = 151841) = 529.452, *p* < .001, Cramer’s *V* = .059). People in the Czech Republic were 1.28 times more likely to die from chronic conditions than people in Slovakia.

**Table 4:** Deaths caused by chronic conditions in CZ and SK 2011

	**Country**		**Total (N,%)**
	**CZ (N,%)**	**SK (N,%)**	
Cancer (C00-C97 and D37-D48)	26101 (26.1%)	12038 (23.2%)	38139 (25.1%)
Cerebrovascular diseases (stroke) (I60-I69)	10244 (10.3%)	5336 (10.3%)	15580 (10.3%)
Heart failure (I50)	4006 (4.0%)	1671 (3.2%)	5677 (3.7%)
Chronic liver disease (K70 and K72-K74)	1899 (1.9%)	1347 (2.6%)	3246 (2.1%)
Chronic obstructive pulmonary disorders (COPD) (J40-J47)	2488 (2.5%)	746 (1.4%)	3234 (2.1%)
Diabetes (E10-E14)	2237 (2.2%)	653 (1.3%)	2890 (1.9%)
Dementia (F00-F03 and G30)	1678 (1.7%)	226 (0.4%)	1904 (1.3%)
Chronic kidney disease (N03-N04, N11-N13 and N18)	767 (0.8%)	488 (0.9%)	1255 (0.8%)
Parkinson's disease (G20-G21)	210 (0.2%)	83 (0.2%)	293 (0.2%)
Multiple sclerosis (G35)	88 (0.1%)	32 (0.1%)	120 (0.1%)
Spinal muscular atrophy and related disorders (G12)	84 (0.1%)	23 (<0.0%)	107 (0.1%)
Neuromuscular disorders (G70-G71)	31 (<0.0%)	13 (<0.0%)	44 (<0.0%)
Acquired immunodeficiency syndrome (AIDS) (B20-B24)	7 (<0.0%)	1 (<0.0%)	8 (<0.0%)
overall proportion of deaths by chronic conditions^b^	49840 (49.9%)	22657 (43.7%)	72497 (47.7%)
overall proportion of deaths by non-chronic conditions	50098 (50.1%)	29246 (56.3%)	79344 (52.3%)
Total	99938 (100%)	51903 (100%)	151841 (100%)

^b^There was a significant difference between proportions of deaths from chronic and non-chronic conditions in CZ and SK, tested by *χ*^2^ Pearson test, p < .001.

Missing N = 2447 (CZ only).

#### Gender and age

Slightly more men than women died from chronic conditions in both CZ (50.8% versus 49.2%) and SK (54.1% versus 45.9%) in 2011. More than 93% of deaths from chronic conditions were in people older than 50 years of age and more than 63% were in people older than 70 years of age (see Table 5). Only in the age group of 51–70 years were more deaths caused by chronic conditions than non-chronic conditions.

**Table 5:** Gender and age distribution of deaths from chronic and non-chronic conditions*

	**Death from chronic condition**				**Total**
	**CZ (N,%)**		**SK (N,%)**		
**Age category**	**Chronic**	**Non-chronic**	**Chronic**	**Non-chronic**	
0-1 years	11 (<0.1)	274 (0.5)	10 (<0.1)	315 (1.1)	610
2-18 years	45 (0.1)	214 (0.4)	45 (0.2)	196 (0.7)	500
19-50 years	2037 (4.1)	3171 (6.3)	1570 (6.9)	2399 (8.2)	9177
51-70 years	17245 (34.6)	11812 (23.6)	8813 (38.9)	7079 (24.2)	44949
71 and older	30502 (61.2)	34627 (69.1)	12219 (53.9)	19257 (65.8)	96605
Total	49840 (100.0)	50098 (100.0)	22657 (100.0)	29246 (100.0)	151841
**Gender**					
Male	25329 (50.8)	25148 (50.2)	12250 (54.1)	14547 (49.7)	77274
Female	24511 (49.2)	24950 (49.8)	10407 (45.9)	14699 (50.3)	74567
Total	49840 (100.0)	50098 (100.0)	22657 (100.0)	29246 (100.0)	151841

*There was a significant association between age category and cause of death (chronic condition yes/no) in both CZ and SK, tested by *χ*2 Pearson test, p < .001. In Slovakia, there was a significant difference between gender and cause of death (p < .001).

#### Place of death

Most of the deaths caused by chronic conditions occurred in hospitals (around 63% in both countries). People in the Czech Republic who died from other conditions were 1.8 times more likely to die at home than people who died from chronic conditions, in Slovakia 1.24 times more (Table 6).

**Table 6:** **Place of deaths from chronic conditions**^**a**^

**Place of death**	**Deaths caused by chronic conditions**				**Total**
	**YES**		**NO**		
	**CZ (N,%)**	**SK (N,%)**	**CZ (N,%)**	**SK (N,%)**	
Home	7672 (15.4)	6232 (27.5)	12372 (24.7)	9333 (31.9)	35609 (23.5)
Hospital	31824 (63.9)	14096 (62.2)	26917 (53.7)	14355 (49.1)	87192 (57.4)
Long term health care facility	7811 (15.7)	719 (3.2)	4410 (8.8)	863 (3.0)	13803 (9.1)
Other	2533 (5.1)	1610 (7.1)	6399 (12.8)	4695 (16.1)	15237 (10.0)
Total	49840 (100.0)	22657 (100.0)	50098 (100.0)	29246 (100.0)	151841 (100.0)

^a^There was a significant association between place of death and cause of death (chronic condition yes/no), tested by *χ*^2^ Pearson test, p < .001.

Missing N = 2447 (CZ only).

### Regression analysis

Only deaths from chronic conditions in people older than 50 years were included in the regression analysis. Cancer patients in both countries were more likely to die at home than patients dying from other chronic conditions. Only patients with heart failure (OR in Czech Rep 1.249, in Slovakia 1.535) and Parkinson’s disease (Slovakia only 2.201) had higher chances of dying at home compared with cancer patients. Women were slightly less likely to die at home in both countries (Czech Rep OR 0.911, *p = .*011, Slovakia 0.879). People who died between the ages of 71–84 years in the Czech Republic were less likely to die at home than younger people (OR 0.849). In Slovakia, people 85 years old and older were most likely to die at home (OR 1.572). There was a contradictory result in the influence of education, when higher education status was associated with higher chance of dying at home in the Czech Republic (OR 1.223) and less chance in Slovakia (OR 0.793). *P* values, odds ratios and confidence intervals for individual factors are shown in Table 7.

**Table 7:** Factors influencing the likelihood of dying from chronic conditions at home*

	**CZ**				**SK**			
	***p***	**OR**	**95% CI for OR**		***p***	**OR**	**95% CI for OR**	
			**Lower**	**Upper**			**Lower**	**Upper**
**Chronic condition**								
Cancer								
Stroke	**<.001**	.651	.591	.716	**<.001**	.666	.608	.729
Dementia	**<.001**	.395	.307	.507	.067	1.341	.980	1.834
COPD	**.008**	.809	.692	.947	**<.001**	.466	.373	.583
Heart failure	**<.001**	1.249	1.116	1.398	**<.001**	1.535	1.353	1.743
Diabetes	.122	1.126	.969	1.308	.076	.826	.669	1.020
Parkinson's disease	.834	.950	.587	1.537	**.001**	2.201	1.360	3.562
Chronic kidney disease	**<.001**	.406	.284	.581	**<.001**	.392	.290	.530
Chronic liver disease	**.001**	.705	.576	.863	**<.001**	.638	.531	.767
Spinal muscular atrophy	.124	1.646	.872	3.107	.583	.734	.243	2.217
Multiple sclerosis	.509	1.283	.612	2.691	.751	.849	.309	2.334
Neuromuscular disorders	.473	.472	.061	3.672	.547	.516	.060	4.434
**Gender**								
Male vs. female	.011	.911	.847	.979	**.001**	.879	.814	.949
**Marital status**								
Single								
Married	.046	1.169	1.003	1.363	.138	1.118	.965	1.295
Divorced	.231	.898	.753	1.071	.050	.826	.682	1.000
Widowed	.761	.975	.828	1.148	.736	1.027	.880	1.198
**Age**								
51-70 years								
71-84 years	**<.001**	.849	.786	.918	.644	1.020	.938	1.109
85 and older	.464	1.040	.937	1.153	**<.001**	1.572	1.399	1.767
**Education**								
Lower vs. higher education^a^	**<.001**	1.223	1.122	1.332	**<.001**	.793	.729	.862

*Binary logistic regression (enter method), sample limited to deaths from chronic conditions (N = 68779) and age of 51 and older.

^a^Missing data on education status CZ 18939 (39.7%), SK 4463 (21.2%), treated by listwise deletion method.

## Discussion

Only 20% and 30% of all deaths in the Czech Republic and Slovakia in 2011 occurred at home. This highlights a major discrepancy between actual and preferred place of death as the majority of people in these countries expressed a preference to die at home (78% in CZ, [17]). This result is similar to other European countries [6,18] and confirms a common trend with more than half of the populations dying in a hospital setting (58.4% in CZ, 54.8% in SK).

Further analysis showed that almost two thirds of patients with chronic conditions died in hospitals in the Czech Republic and Slovakia. This number is considerably higher than in other countries with similar sized populations, such as the Netherlands [2], where only around 30% of deaths in people with chronic conditions occur in a hospital setting. Regression analysis confirmed that place of death is strongly associated with underlying cause of death with cancer patients being more likely to die at home than patients dying from other chronic conditions.

The results of this study support several trends identified in other countries, such as the discovery that more than half of the population die in hospitals and that men are more likely to die at home than women, probably because they die at a younger age when their wives or partners can help facilitate care at home [6,10,18,19]. However, we also found some differences between the Czech Republic and Slovakia and other countries. In the Czech Republic and Slovakia most people who died from chronic conditions in 2011 died in hospitals (around 63% in both countries). There are several possible explanations for this finding. In the Netherlands, where only a third of such patients die in hospitals, nursing home care is developed to a very high level and provides care for similar proportion of dying people as hospitals [2]. In neither the Czech Republic nor Slovakia are such nursing homes available. Care homes for older people or local variations of nursing homes usually do not have a physician on the staff and many GPs do not have enough experience with symptom management at the end of life [20]. When complications occur the patient is most likely to be transported to hospital. Institutes for long term patients, which do have physicians on the staff, are often under such budgetary pressures that they can’t afford for example appropriate analgesics (Slama O, unpublished presentation^a^).

Odds of dying at home are increased when home palliative care services are available [13]. There are only few palliative home care services available in the Czech Republic and none in Slovakia [21]. The reason for this might be the fact that palliative home care is still not recognized in the health care insurance law and hence the palliative home care services can’t access the governmental health care budget. Legislative issues are main barriers in the further development in both countries, with long term care (including hospice care) being especially challenging area where two sectors, health and social care, are involved and coordination of their policies is difficult [22-24]. The housing situation in the Czech Republic and Slovakia is also an important factor which influences the potential care for a dying relative at home. Typical home environments currently available are either two bedroom flat in cities or larger family houses in the countryside which means that when children want to provide care for their elderly parents they have to struggle either with space limitations or availability of support services which are rarely provided in rural areas [24].

There was a contradictory effect of education found in the selected countries. In the Czech Republic, people with higher education were more likely to die from chronic conditions at home (OR 1.223) while in Slovakia this was less likely (OR 0.793). In other countries the effect between greater education and chance of dying at home is usually positive [6,10,25]. Possible explanation for the difference in Slovakia might be the trend of massive urbanization in 1970’s and 1980’s with higher educated people typically moving into small flats and consequently being less likely to access and provide informal care at their homes. However, recent trends show that older people with university education tend to move out of cities [26], so this association has to be further explored.

This study also has its limitations. Firstly, not all variables, relevant to predicting place of death were available [1,25], for example socio-economic status or level of urbanization. Problems with cause of death coding in death certificates is another well-known issue [27,28] and has been reported also from both selected countries [29,30]. A specific complication is the coding of place of death. Apart from lacking an universally accepted coding system [1] the practice of completing this question in forms is not unified. One example of confused practice is when death in social care home is coded as death at home. This situation sometimes happens when the patient has the address of the care home registered as his or her home address. It is not known how often it is, but bias of up to several percentages is possible in the Czech Republic [31]. In Slovakia, this category is not available at all. Similar confusion can occur in coding deaths in institutes for long term patients, which are sometimes mixed with acute hospitals, especially when it was only a long term care ward within a larger hospital. Palliative care units or hospices are not included in either of Czech and Slovak certificates and the coding of hospice deaths is also not clear as hospices can be recognized either as social care homes or “other”.

## Conclusions

Analysis of Czech and Slovak death certificates data pointed out several trends previously identified in other countries. More than half of the population dies in hospitals, although the preferred place of death is home for most people. Patients with cancer or heart failure have better chances to die at home than patients dying from other chronic conditions. Apart from cause of death, other sociodemographic variables like gender, age or education influence the place of death. Czech and Slovak patients who died from chronic conditions were more likely to die in hospital setting than patients in other countries. This finding should be taken into account by policy makers and potential changes in health care services delivery suggested, for example with regards of end-of-life care education for general practitioners or the role of nursing homes. Support for palliative care home teams could also improve the likelihood to balance the preferred and actual place of death. However, more research is needed to understand what are the motives for place of death preferences, what conditions at home would really meet people’s expectations, as well as to explore the complex system of influencing factors in more detail.

## Endnote

^a^Slama O: Improving Access to Pain Medicines: the example of the Czech Republic. Presentation during 13th World Congress of the European Association for Palliative Care; Prague; 1st June, 2013.

## Abbreviations

CZ: The Czech Republic; SK: Slovakia; CI: Confidence interval; OR: Odds ratio; ICD-10: International classification of diseases, 10th edition.

## Competing interests

The authors declared that they have no competing interests.

## Authors’ contributions

ML obtained the data and performed the analysis. The manuscript was drafted by ML and approved by SB and SP. All three authors read and approved the final manuscript.

## Pre-publication history

The pre-publication history for this paper can be accessed here:

http://www.biomedcentral.com/1472-684X/13/13/prepub
